# Prevalence of Orthostatic Autonomic Dysregulation in Pediatric Concussion

**DOI:** 10.1001/jamanetworkopen.2025.22309

**Published:** 2025-07-22

**Authors:** Veronik Sicard, Tenaaz Irani, Andrée-Anne Ledoux, Ivan Terekhov, Richard J. Webster, Ewa Sucha, Stephen A. Kutcher, Lauren Xinyue Duan, Farzaneh Dashti, Achelle Cortel-Leblanc, John Leddy, Lawrence Richer, Nick Reed, Kim Connelly, Charlotte Anderson, Sharon Johnston, Roger Zemek

**Affiliations:** 1 Children’s Hospital of Eastern Ontario Research Institute, Ottawa, Ontario, Canada; 2 Faculty of Medicine, University of Ottawa, Ottawa, Ontario, Canada; 3 Department of Neuroscience, Carleton University, Ottawa, Ontario, Canada; 4 Department of Cellular and Molecular Medicine, University of Ottawa, Ottawa, Ontario, Canada; 5 360 Concussion Care, Ottawa, Ontario, Canada; 6 Institut du Savoir Montfort, Ottawa, Ontario, Canada; 7 Division of Neurology, Department of Medicine, Queensway Carleton Hospital, Ottawa, Ontario, Canada; 8 University at Buffalo Medical Doctors, Department of Orthopaedics and Sports Medicine, Jacobs School of Medicine and Biomedical Sciences, State University of New York at Buffalo, Buffalo, New York; 9 Women and Children’s Health Research Institute, College of Health Sciences, University of Alberta, Edmonton, Alberta, Canada; 10 Department of Occupational Science and Occupational Therapy, University of Toronto, Toronto, Ontario, Canada; 11 Keenan Research Center for Biomedical Science, Li Ka Shing Knowledge Institute, St Michael’s Hospital, Unity Health Toronto, University of Toronto, Toronto, Ontario, Canada; 12 Division of Cardiology, St Michael’s Hospital, Unity Health Toronto, University of Toronto, Toronto, Ontario, Canada; 13 Alpha Health Services, Toronto, Ontario, Canada; 14 Department of Physiotherapy, University of Toronto, Ontario, Canada; 15 Department of Family Medicine, University of Ottawa, Ottawa, Ontario, Canada; 16 Department of Pediatrics, Faculty of Medicine, University of Ottawa, Ottawa, Ontario, Canada; 17 Faculty of Medicine and Dentistry, College of Health Sciences, University of Alberta, Edmonton, Alberta, Canada

## Abstract

**Importance:**

Pediatric concussion can affect the autonomic nervous system. Understanding the prevalence and characteristics of physiological autonomic dysregulation (AD) and symptom provocation following concussion is crucial for optimizing recovery and developing targeted interventions.

**Objectives:**

To determine the prevalence of AD and symptom provocation upon postural change and to (1) explore the overlap between AD and symptom provocation, (2) apply adult orthostatic tachycardia criteria (heart rate [HR] ≥30 bpm) to this pediatric population, (3) explore associations of demographic and injury characteristics with AD and symptom provocation, and (4) characterize AD presentation and identify the predominant orthostatic sign.

**Design, Setting, and Participants:**

This retrospective cohort study used electronic medical record review of clinical data at tertiary care concussion clinics that operate as a learning health system in Canada. Patients aged 5 to younger than 18 years who presented to those clinics within 45 days of a diagnosed concussion between August 2022 and January 2024 were included. Analysis was conducted from April to October 2024.

**Exposure:**

Diagnosis of concussion.

**Main Outcomes and Measures:**

Primary outcomes were AD presence (defined as the presence of either a decrease in systolic BP [≥20 mm Hg], decrease in diastolic BP [≥10 mm Hg], or increase in HR [≥40 bpm]), and symptom provocation (new or worsening). HR, blood pressure (BP), and symptoms were measured after a 2-minute supine rest and 1 minute of unsupported standing.

**Results:**

Of 764 eligible patients, 451 (231 female [51.22%]; 129 [28.60%] aged 5-11 years and 322 [71.40%] aged 12 to <18 years) were included; objective AD was identified in 45 patients (9.98%; 95% CI, 7.54%-13.09%), primarily by HR changes (22 of 45 patients [48.89%]). When applying adult tachycardia criteria, AD prevalence was 23.73% (95% CI, 20.03%-27.88%). Orthostatic symptom provocation occurred in 103 of 445 patients (23.15%; 95% CI, 19.47%-27.28%). Among 134 patients with either AD or symptom provocation, 12 (8.96%) exhibited both. Patients with AD had higher rates of neurodevelopmental disorders than patients without AD (15 of 45 patients [33.33%] vs 75 of 406 patients [18.47%]; *P* = .02), while symptom provocation was associated with female sex (63 of 103 patients [61.17%] vs 166 of 342 patients [48.54%]; *P* = .03), preexisting mental health disorders (28 of 103 patients [27.18%] vs 45 of 342 patients [13.16%]; P < .001), higher concussion symptom burden (median [IQR] symptom intensity score, 46.00 [16.00-67.00] vs 21.00 [6.00-46.00]; *P* < .001), and status of posttraumatic amnesia at injury (22 of 103 patients with posttraumatic amnesia [22.68%] vs 63 of 342 patients without [18.92%]; *P* = .009).

**Conclusions and Relevance:**

In this cohort study of children and adolescents with concussion, approximately 1 in 10 exhibited AD and 1 in 4 exhibited symptom provocation. The observed low concordance between physiological AD and symptom provocation, along with their distinct clinical profiles, might suggest these represent separate phenomena in pediatric concussion; future research should explore whether incorporating both measures into clinical assessments enhances understanding of concussion and informs targeted interventions to optimize recovery.

## Key Points

**Question:**

What is the prevalence and clinical presentation of autonomic dysregulation (AD), characterized by orthostatic hypotension, tachycardia, and symptom provocation, within 45 days of a pediatric concussion?

**Findings:**

In this cohort study using medical record review of 451 pediatric patients diagnosed with concussion, 9.98% showed physiological evidence of AD and 23.15% reported symptom provocation during postural change. Physiological AD was not always accompanied by subjective symptom reporting.

**Meaning:**

This study challenges existing clinical assumptions about pediatric concussion management and reinforces the need for objective physiological testing in concussion assessment.

## Teaser

This cohort study examines the prevalence of autonomic dysregulation and symptom provocation upon postural change among children and adolescents with concussion.

## Introduction

One in 10 students in grades 6 to 10 in Canada reported experiencing a concussion in 2019, primarily through sports (59.1%).^1^ Concussion symptoms include headaches, anxiety, dizziness, sleep disturbances, and photosensitivity. Concussion may disrupt autonomic function through a catecholamine release and inflammatory response to the brain injury.^2^ Elevated sympathetic activity results in autonomic dysregulation (AD) characterized by systemic effects such as unstable heart rate (HR), fluctuating blood pressure (BP), and symptoms that overlap with the typical postconcussion presentations.^3,4,5,6,7^ These symptoms include headache, lightheadedness, nausea, dizziness, photophobia and phonophobia, and anxiety, and may both arise from and contribute to concussion-related symptoms. While concussion is the second leading trigger of pediatric postural orthostatic tachycardia syndrome (POTS),^7^ a chronic AD condition affecting approximately 1% of youths worldwide,^8^ the association of AD with concussion is poorly understood due to limited research and inconsistent AD definitions used across studies.^9,10,11,12,13,14,15,16^ Understanding AD prevalence and characteristics postconcussion is crucial because targeted early intervention could improve children’s long-term outcomes.

Pediatric concussion studies report AD prevalence, characterized by orthostatic tachycardia or hypotension, ranging from 3% to 73%, with variations due to differences in orthostatic challenges and metrics, study populations, and end points.^10,12,13,14,15,16^ Beyond objective AD measures, it is crucial to consider orthostatic symptom provocation, with studies reporting headaches, lightheadedness, or nausea in 37% to 47% of participants upon standing, although the time since injury varied.^9,11,15^ The AD prevalence is further complicated by different outcome measures, with some studies focusing solely on vital signs,^13,16^ others on symptoms,^9,12^ and some on specific metrics like tachycardia^12^ or hypotension,^11^ with or without concurrent symptom monitoring. These studies are limited by small sample sizes^10,12,13,15^ and/or inclusion of patients in the subacute, chronic, or different phases after injury.^10,12,13,14,15^

The primary objective of this study was to determine the prevalence of (1) physiological AD characterized by orthostatic hypotension and tachycardia and (2) orthostatic symptom provocation in children and adolescents within 45 days of a concussion. Secondary objectives were to (1) determine the prevalence of AD and symptom provocation stratified by age groups (5-11 years and 12-18 years) and sex, (2) explore the overlap between AD and symptom provocation, (3) apply the adult orthostatic tachycardia threshold (HR ≥30 bpm)^17^ to this pediatric population to assess how this commonly used threshold may influence prevalence estimates and facilitate comparison with previous studies, (4) explore the association of demographic and injury variables with the presence of AD and symptom provocation, and (5) characterize AD presentation and identify the predominant orthostatic sign.

## Methods

### Study Design

This study is a retrospective cohort study, specifically an electronic medical record (EMR) review study of clinical data collected within a network of concussion clinics (Ottawa, Mississauga, and Toronto, Canada) operating as a learning health system and providing care to patients with acute to persistent symptoms of all ages and mechanisms of injury. The study followed the Reporting of Studies Conducted Using Observational Routinely Collected Data (RECORD) recommendations^18^ and was reported under the Strengthening the Reporting of Observational Studies in Epidemiology (STROBE) reporting guideline.^19^ This project was approved by the Children’s Hospital of Eastern Ontario research ethics board. All participants gave passive consent for research use of deidentified clinical data via an acknowledgment statement read during initial clinical registration, explaining potential research use of standard care data following research ethics board approval, with opt-out provisions available.

### Participants

Data were extracted from the EMR using Looker Studio (Google) analytics for patients aged 5.00 to 17.99 years who presented to clinics between August 12, 2022, and January 2, 2024, within 45 days of a diagnosed concussion and without cardiovascular problems. The analytics team applied filters for age, injury timeframe, and cardiovascular history, and generated a data cut containing all relevant study variables for eligible participants, which was securely transferred to the research team. Records were manually reviewed to confirm eligibility, with additional exclusions for Glasgow Coma Scale score (≤13), brain injuries requiring neurosurgical intervention, clinically significant neurological disease affecting autonomic function (eg, structural cerebral abnormalities, cerebral palsy, congenital central hypoventilation, Prader-Willi syndrome, familial dysautonomia, spinal cord lesions, and peripheral neuropathy), severe preexisting neurological developmental delay affecting communication, and missing orthostatic vital signs data.

### Procedures

Patient data were collected through self-reported questionnaires completed by participants or caregivers before their clinic visit, or by physicians and trained research assistants during the visit. Questionnaires included demographic, medical, and injury information, and the Post-Concussion Symptom Scale from the Sport Concussion Assessment Tool to measure symptom burden (22 symptoms rated 0-6; range, 0-132).^20^ Orthostatic vital signs were recorded by physicians or research assistants at the start of the visit, but testing was discretionary (eg, omitted for patients unable to stand unassisted due to concurrent orthopedic injury or due to clinic flow or time constraint).

### Orthostatic Vital Signs and Symptom Testing

BP and HR were measured after a 2-minute supine rest and again after approximately 1 minute standing unsupported, consistent with clinical practice,^21^ using medical-grade automatic BP monitors (Welch Allyn ProBP line). Orthostatic symptoms were systematically assessed and recorded in both the supine and standing positions during the vital signs testing. Patients were directly asked about the presence of any of the following symptoms: blurry vision, confusion, dizziness, headache, lightheadedness, nausea, presyncope, or weakness.

### Outcome Measures

Primary outcomes were the prevalence of physiological AD and orthostatic symptom provocation upon postural change. AD was defined by meeting any one consensus-defined criterion upon standing: (1) a decrease in systolic BP (SBP) of 20 mm Hg or greater,^17,22^ (2) a decrease in diastolic BP (DBP) of 10 mm Hg or greater,^17,22^ or (3) an increase in HR of 40 bpm or more.^17,23,24^ Although consensus definitions of orthostatic hypotension and tachycardia apply only to patients aged 12 years and older due to limited research in younger populations,^17^ we included younger children as an exploratory cohort. Orthostatic symptom provocation was defined as new or worsening symptoms upon standing.

Secondary outcomes included the prevalence of AD and orthostatic symptom provocation stratified by age groups (5 to 11 years and 12 to <18 years) and sex (female or male), prevalence of AD based on the adult criteria for orthostatic tachycardia (HR ≥30 bpm),^17^ and orthostatic changes in SBP, DBP, and HR as continuous variables (rather than categorized as the presence or absence of AD and symptom provocation). Previous studies indicated that many adolescents with concussion and orthostatic symptoms exhibit HR increases of 30 to 39 bpm during head-up tilt table or active standing tests,^14,15^ which are below the cutoff for their age group.

### Statistical Analyses

Descriptive statistics summarized patient demographics, medical histories, injury characteristics, and AD and symptom provocation characteristics. Wilson scores with 95% CIs described the overall prevalence of AD and orthostatic symptom provocation, as well as stratified by age group and sex. Descriptive analysis also examined overlap between AD and symptom provocation, as well as the occurrence of AD criteria (individually and combined) among AD cases. Orthostatic vital sign changes were summarized using medians and IQRs for AD and non-AD groups, as well as by age group.

Univariate models (Wilcoxon rank sum test, Pearson χ^2^ test, or Fisher exact test, as appropriate) explored associations of demographics and injury characteristics with both AD and symptom provocation. No corrections for multiple comparisons were applied due to the exploratory nature of the analysis.

In all analyses, a 2-sided *P* < .05 was considered significant. Analyses were performed using R statistical software v4.3.1 (R Project for Statistical Computing) from April to October 2024.^25^

## Results

### Participant Characteristics

Data from 764 pediatric patients were extracted from the EMR. After excluding 6 for presenting 45 days or more postinjury, 14 for a history of cardiovascular or autonomic problems, and 293 for missing orthostatic vital signs (eFigure in Supplement 1), 451 patients (231 female [51.22%]; 129 aged 5-11 years [28.60%]; 322 aged 12 to <18 years [71.40%]) were included in the analyses. Demographic and injury characteristics are presented in Table 1, and a comparison between included and excluded patients is provided in eTable 1 in Supplement 1. Six patients had missing symptom data, yielding 445 patients for symptom provocation analyses.

**Table 1.** Participant Demographics, Medical History, and Injury Characteristics

Variables	Participants, No. (%)		
	Full sample (N = 451)	5-11 y (n = 129)	12 to <18 y (n = 322)
Age, median (IQR), y	14.00 (12.00-15.00)	10.00 (9.00-12.00)	15.00 (14.00-16.00)
Sex			
Female	231 (51.22)	61 (47.29)	170 (52.80)
Male	220 (48.78)	68 (52.71)	152 (47.20%)
Statistics Canada physical activity guidelines^a^			
Met guidelines	182 (44.28)	29 (22.83)	153 (53.87)
Missing, No.	40	2	38
Medical history			
Neurological disorders	13 (2.88)	4 (3.10)^b^	9 (2.80)^c^
Migraines	33 (7.32)	8 (6.20)	25 (7.76)
Attention deficit/hyperactivity disorder	69 (15.30)	12 (9.30)	57 (17.70)
Learning disability	50 (11.09)	6 (4.65)	44 (13.66)
Other neurodevelopmental disorder	6 (1.33)	2 (1.55)	4 (1.86)
Anxiety	58 (12.86)	7 (5.43)	51 (15.84)
Depression	28 (6.21)	0	28 (8.70)
Previous concussion	163 (36.14)	33 (25.58)	130 (40.37)
Longest previous concussion duration			
<4 wk	95 (58.28)	19 (57.58)	76 (58.46)
≥4 wk	40 (24.54)	8 (24.24)	32 (24.62)
Unknown	28 (17.18)	6 (18.18)	22 (16.92)
Medications			
Stimulants	36 (7.98)	4 (3.10)	32 (9.94)
Antidepressants	38 (8.43)	4 (3.10)	34 (10.56)
Other	4 (0.89)^d^	0	4 (1.24)^d^
Injury-related characteristics			
Concussion mechanism			
Sport and recreation	318 (70.51)	83 (64.34)	235 (72.98)
Non–sport-related	106 (23.50)	40 (31.01)	66 (20.50)
Motor vehicle collision	14 (3.10)	1 (0.78)	13 (4.04)
Assault	13 (2.88)	5 (3.8)	8 (2.48)
Loss of consciousness			
Yes	52 (11.82)	7 (5.56)	45 (14.33)
Missing, No.	11	3	8
Posttraumatic amnesia			
Yes	103 (23.68)	23 (18.85)	80 (25.56)
Missing	16	7	9
Seizure			
Yes	6 (1.38)	1 (0.79)	5 (1.61)
Missing, No.	15	3	12
Confusion			
Yes	172 (39.27)	40 (32.26)	132 (42.04)
Missing, No.	13	5	8
Answering questions slowly			
Yes	93 (21.14)	23 (18.56)	70 (22.15)
Missing, No.	11	5	6
Time since concussion, median (IQR), d	8.00 (4.00-15.00)	8.00 (4.00-15.00)	8.00 (4.00-15.00)
Symptom intensity at presentation, median (IQR) score	23.00 (8.00-51.00)	11.00 (3.00-22.00)	37.00 (11.00-62.00)

^a^ At least 60 minutes per day of moderate to vigorous aerobic physical activities.

^b^ Epilepsy or seizures (2 participants), meningitis (1 participant), and chronic fatigue syndrome (1 participant).

^c^ Epilepsy or seizures (2 participants), spinal cord issues (2 participants), abdominal migraines (1 participant), past brain tumor (1 participant), carpal tunnel syndrome (1 participant), chronic fatigue syndrome (1 participant), and stroke (1 participant).

^d^ Atypical antidepression (1 participant), atypical antipsychotic (1 participant), and attention deficit/hyperactivity disorder and/or anxiety medication unspecified (1 participant).

### Primary Results: Prevalence of AD and Symptom Provocation

Overall AD prevalence was 9.98% (95% CI, 7.55%-13.09%; 45 of 451 patients). Symptom provocation was reported in 23.15% (95% CI, 19.47%-27.28%) of patients (103 of 445 patients).

### Secondary Results

#### Prevalence of AD and Symptom Provocation Stratified by Age and Sex

Small sample sizes in each group precluded statistical comparisons of AD or symptom provocation prevalence across age and sex. AD prevalence appeared highest among males aged 12 to younger than 18 years (15.13%; 95% CI, 10.30%-21.68%), and lowest in males aged 5 to 11 years (7.25%; 95% CI, 3.18%-16.09%). AD prevalence among females was 11.48% (95% CI, 5.67%-21.84%) among those aged 5 to 11 years and 5.88% (95% CI, 3.23%-10.49%) among those aged 12 to younger than 18 years (eTable 2 in Supplement 1). Symptom provocation prevalence appeared higher among females (26.67% [95% CI, 17.13%-39.01%] in those aged 5 to 11 years and 27.81% [95% CI, 21.61%-35.00%] in those aged 12 to <18 years) than males (16.67% [95% CI, 9.57%-27.43%] in those aged 5 to 11 years and 19.33% [95% CI, 13.81%-26.39%] in those aged 12 to <18 years) (eTable 2 in Supplement 1).

#### Prevalence of AD Using the Adult Tachycardia Criteria and Concordance Between AD and Symptom Provocation

The overall AD prevalence was 23.73% (95% CI, 20.03%-27.88%; 108 of 451 patients) when applying the adult tachycardia criteria. Limited overlap was found between AD and symptom provocation. Of the 134 patients with either condition, 12 (8.96%) exhibited both AD and symptom provocation. Most cases fell into 2 distinct categories: 31 of 134 patients (23.13%) showed physiological evidence of AD without symptom provocation, while 91 of 134 patients (67.91%) reported symptom provocation despite not meeting the formal clinical criteria for AD.

#### Association of AD or Symptom Provocation With Key Variables

A higher proportion of children with AD had a prior neurodevelopmental disorder compared with those without AD (15 of 45 patients [33.33%] vs 75 of 406 patients [18.47%]; *P* = .02) (Table 2). Among those with symptom provocation, a greater proportion was female (63 of 103 patients [61.17%] vs 166 of 342 patients [48.54%]; *P* = .03) and had a history of mental health disorder (28 of 103 patients [27.18%] vs 45 of 342 patients [13.16%]; *P* < .001) (Table 2). Concussion symptom burden was also higher in those with symptom provocation compared with those without (median [IQR] symptom intensity score, 46.00 [16.00-67.00] vs 21.00 [6.00-46.00]; *P* < .001). Prevalence of posttraumatic amnesia also significantly differed between patients with symptom provocation (22 patients [22.68%] with posttraumatic amnesia; 53 patients without posttraumatic amnesia [54.64%]; and 22 patients with unsure status [22.68%]) and without symptom provocation (63 patients with posttraumatic amnesia [18.92%]; 231 patients without posttraumatic amnesia [69.37%] and 39 with unsure status [11.71%]) (*P* = .009).

**Table 2.** Comparison of Participant Characteristics by AD and Orthostatic Symptom Provocation Status

Characteristic	AD, No. (%) (n = 45)	No AD, No. (%) (n = 406)	*P *value	Symptom provocation, No. (%) (n = 103)	No symptom provocation, No. (%) (n = 342)	*P *value
Age, median (IQR, y)	14.00 (12.00-15.00)	14.00 (12.00-15.00)	.51	14.00 (12.00-15.00)	14.00 (12.00-15.00)	.66
Sex						
Female	17 (37.78)	214 (52.71)	.06	63 (61.17)	166 (48.54)	.03
Male	28 (62.22)	192 (47.29)		40 (38.83)	176 (51.46)	
Statistics Canada physical activity guidelines^a^						
Met guidelines	21 (53.85)	161 (43.28)	.21	36 (40.45)	144 (45.57)	.39
Missing, No.	6	34	NA	14	26	NA
Medical history						
Migraines	1 (2.22)	32 (7.88)	.23	5 (4.85)	28 (8.19)	.26
Neurodevelopmental disorder^b^	15 (33.33)	75 (18.47)	.02	19 (18.45)	70 (20.47)	.65
Mental health disorder^c^	6 (13.33)	67 (16.50)	.58	28 (27.18)	45 (13.16)	<.001
Previous concussion	20 (44.44)	143 (35.22)	.22	38 (36.89)	124 (36.26)	.91
Concussion mechanism						
Sport or recreational	35 (77.78)	283 (69.70)	.41	65 (63.11)	249 (72.81)	.11
Non–sport-related^d^	8 (17.78)	108 (26.60)		32 (31.07)	82 (23.98)	
Unknown	2 (4.42)	15 (3.69)		6 (5.83)	11 (3.22)	
Loss of consciousness						
Yes	10 (22.22)	42 (10.63)	.06	16 (16.16)	35 (10.45)	.18
No	33 (73.33)	312 (78.99)		71 (71.72)	269 (80.30)	
Unsure	2 (4.44)	41 (10.38)		12 (12.12)	31 (9.25)	
Missing, No.	0	11	NA	4	7	NA
Posttraumatic amnesia						
Yes	8 (18.18)	78 (19.90)	.46	22 (22.68)	63 (18.92)	.009
No	27 (61.36)	261 (66.58)		53 (54.64)	231 (69.37)	
Unsure	9 (20.45)	53 (13.52)		22 (22.68)	39 (11.71)	
Missing, No.	1	14	NA	6	9	NA
Confusion						
Yes	18 (41.86)	154 (38.99)	.56	45 (45.00)	125 (37.54)	.35
No	21 (48.84)	180 (45.57)		40 (40.00)	159 (47.75)	
Unsure	4 (9.30)	61 (15.44)		15 (15.00)	49 (14.71)	
Missing, No.	2	11	NA	3	9	NA
Answering questions slowly						
Yes	14 (31.11)	132 (33.42)	.72	39 (39.00)	106 (31.74)	.13
No	23 (51.11)	178 (45.06)		37 (37.00)	162 (48.50)	
Unsure	8 (17.78)	85 (21.52)		24 (24.00)	66 (19.76)	
Missing, No.	0	11	NA	3	8	NA
Time since concussion, median (IQR), d	6.00 (4.00-14.00)	8.00 (4.00-15.00)	.42	7.00 (4.00-15.00)	8.00 (4.00-14.00)	.83
Symptom intensity at presentation						
Median (IQR) score	39.00 (9.00-52.00)	23.00 (8.00-51.00)	.32	46.00 (16.00-67.00)	21.00 (6.00-46.00)	<.001
Missing	2	3	NA	1	3	NA

Abbreviations: AD, autonomic dysregulation; NA, not applicable.

^a^ At least 60 minutes per day of moderate to vigorous aerobic physical activities.

^b^ Neurodevelopmental disorders combine attention-deficit disorder with or without hyperactivity, learning disabilities, and other conditions reported.

^c^ Mental health disorders combine depression and anxiety.

^d^ Non–sport-related concussions include those sustained outside of athletic activities, including incidents such as motor vehicle collisions, assaults, falls, and other non-sport–related accidents.

#### Characteristics of AD

The most frequently met AD criterion was HR (22 of 45 patients [48.89%]), followed by SBP (17 of 45 patients [37.78%]) and DBP (10 of 45 patients [22.22%]). Most patients met a single criterion (41 of 45 patients [91.11%]), while none met all 3 criteria. As shown in Table 3, HR increase alone accounted for the largest proportion of AD cases (19 of 45 patients [42.22%]), followed by isolated SBP decrease (16 of 45 patients [35.56%]) and DBP decrease (6 of 45 patients [13.33%]). Only a small subset met combinations of criteria (4 of 45 patients [8.89%]).

**Table 3.** Frequency of Meeting a Single or Multiple Criteria for AD

Pattern	AD criteria^a^			Participants, No. (%) (N = 45)
	Decrease in SBP ≥20 mm Hg	Decrease in DBP ≥10 mm Hg	Increase in HR ≥40 bpm	
1	Not met	Not met	Met	19 (42.22)
2	Met	Not met	Not met	16 (35.56)
3	Not met	Met	Not met	6 (13.33)
4	Not met	Met	Met	3 (6.67)
5	Met	Met	Not met	1 (2.22)

Abbreviations: AD, autonomic dysregulation; DBP, diastolic blood pressure, HR, heart rate; SBP, systolic blood pressure.

^a^ AD was defined as the presence of at least 1 of these 3 signs upon standing.

**Figure 1.**
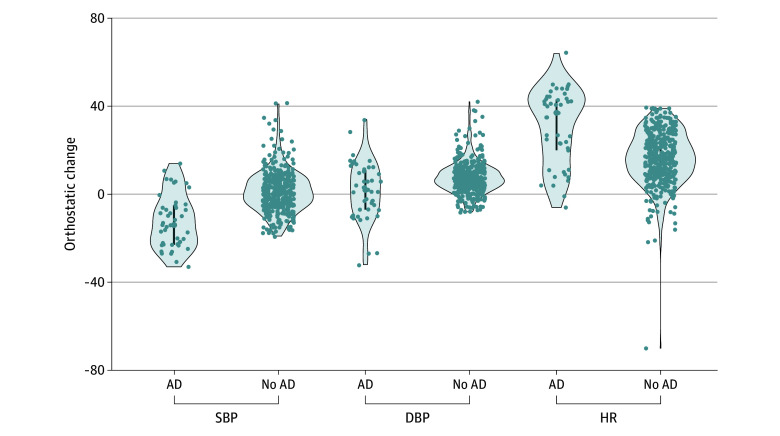
Orthostatic Change in Vital Signs During a 1-Minute Active Standing Test Each violin plot represents orthostatic change in systolic blood pressure (SBP), diastolic blood pressure (DBP), and heart rate (HR) in children who met criteria for autonomic dysregulation (AD; presence of at least 1 of these 3 symptoms upon standing: decrease in SBP ≥20 mm Hg, decrease in DBP ≥10 mm Hg, or increase in HR ≥40 bpm) and who did not meet criteria. Each violin plot represents the distribution of the variable, with the width of the plot showing the density of data points at different values; dots represent individual data points while the blue line in the center represents the IQR of the median.

The observed median (IQR) orthostatic changes in the overall sample were 0.00 mmHg (−6.00 to 6.00 mmHg) for SBP, 7.00 mmHg (3.00 to 11.00 mmHg) for DBP, and 18.00 bpm (9.00 to 27.00 bpm) for HR (eTable 2 and eTable 3 in Supplement 1). Patients classified with AD seemed to exhibit larger HR orthostatic increase upon standing (median [IQR], 37.00 [20.00 to 43.00] bpm) compared with those without AD (median [IQR], 17.00 [9.00 to 25.00] bpm) (eTable 3 in Supplement 1 and Figure 1). SBP median (IQR) orthostatic change was −14.00 mmHg (−23.00 to −5.00 mmHg) in the AD group vs a slight increase of 1.00 mmHg (−5.00 to 7.00 mmHg) in the non-AD group (eTable 3 in Supplement 1 and Figure 1). In contrast, DBP responses were most similar between groups, with a median (IQR) of 2.00 mmHg (−7.00 to 11.00 mmHg) for the AD group and 7.00 mmHg (3.00 to 11.00 mmHg) for the non-AD group (eTable 3 in Supplement 1 and Figure 1).

### Post Hoc Exploratory Analysis: Characteristics of Symptom Provocation

**Figure 2.**
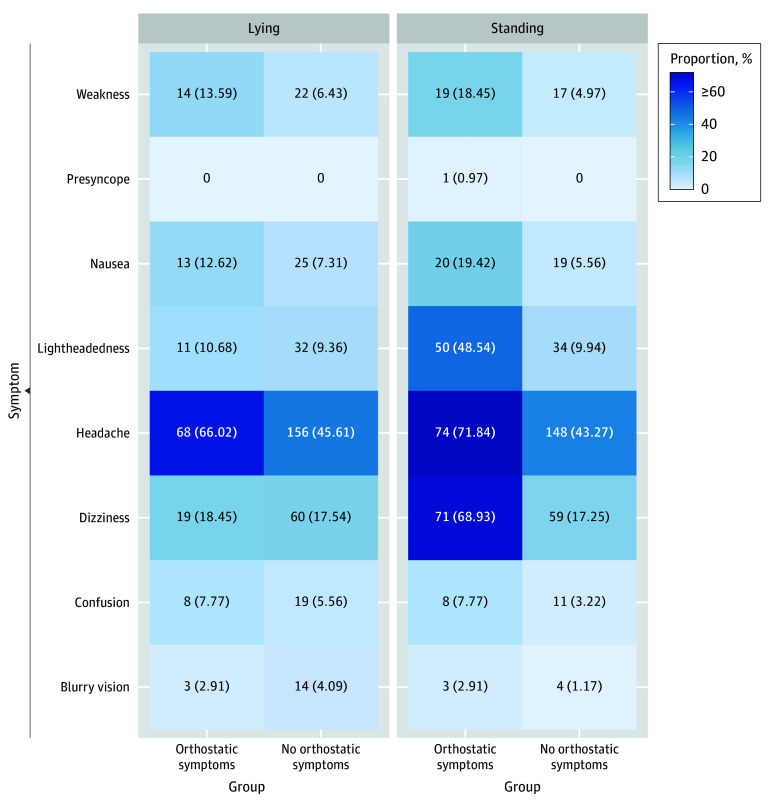
Symptoms Provoked During the Orthostatic Challenge Heatmap of the proportion of symptoms (No. [%]) by group (patients with symptom provocation following postural change vs those without symptom provocation) and position (lying vs standing) during the 2-minute active standing test. Of 451 participants, 6 participants were not included in this figure due to missing orthostatic symptom data.

A heatmap was generated to examine the distribution of symptoms in participants with and without symptoms in the supine and standing positions (Figure 2). Participants with symptom provocation on postural change appeared to report higher baseline headache rates (68 of 103 participants [66.02%] vs 156 of 342 participants [45.61%]), although no statistical analyses were conducted given the limited sample size and exploratory nature of this analysis. Among those reporting symptom provocation, lightheadedness (ranging from 11 of 103 participants [10.67%] to 50 of 103 participants [48.54%]) and dizziness (ranging from participants 19 of 103 participants [18.45%] to 71 of 103 participants [68.93%]) were most common, while presyncope and blurry vision were rare (ranging from 0 participants to 3 of 103 participants [2.91%]).

## Discussion

This cohort study using EMR review found that physiological AD manifested distinctly from symptom provocation in pediatric concussion. While approximately 1 in 10 patients met criteria for orthostatic tachycardia and hypotension, 1 in 4 reported symptom provocation upon postural change, with limited overlap between patients. This minimal overlap, along with differing clinical profiles challenges the assumption that orthostatic symptoms reliably indicate underlying AD. Moreover, patients with symptom provocation had higher concussion symptom burden than those without, suggesting that provocation reflects general postconcussion effects rather than primary autonomic disturbances.

The AD prevalence in this sample aligns with findings from a study of patients aged 8 to 25 years presenting to a specialty concussion clinic.^14^ In contrast, studies focusing on adolescents reporting AD-related symptoms 2 to 120 days postinjury reported higher rates—approximately one-fifth of adolescents met POTS diagnostic criteria (HR increase ≥40 bpm without hypotension) during a 1-minute active standing test,^10^ while nearly one-third met POTS or neurally mediated hypotension criteria (SBP decrease ≥25 mm Hg after 3 minutes of standing) during a 10-minute active standing test.^15^ These discrepancies likely reflect sampling and methodological differences. Prior studies included participants with AD-related symptoms, creating a predisposed sample, whereas our cohort represented a broader concussion population. Longer test durations also tend to capture more cases due to progressive HR increases.^26^ Head-up tilt table testing has shown even higher prevalence rates, with approximately 7 in 10 patients with pediatric concussion testing positive using varied criteria.^12,13^ While tilt table tests are more sensitive than active standing for detecting POTS,^27,28^ they may also reduce specificity, increasing false positives. Age-related differences were observed, with children showing lower AD prevalence than adolescents. This likely reflects limitations of consensus thresholds, which were developed for adolescents aged 12 years and older, emphasizing the need for age-specific reference ranges from typically developing children. Applying the adult tachycardia criterion approximately doubled the AD prevalence in our cohort, consistent with other concussion studies using an active standing test.^14,16^ Another study found that nearly two-thirds of youths who did not meet tachycardia criteria exhibited HR increases just below the diagnostic threshold.^15^ These intermediate responses may reflect AD rather than an overt dysfunction. The clinical significance of these intermediate findings warrants further investigation. While not intended as a diagnostic cutoff in this context, applying the adult threshold provides useful context for comparing findings across studies and highlights the need for age-specific normative data.

In this study, approximately 25% of patients experienced symptom provocation, which aligns with previous studies, but is slightly lower than reported prevalence rates. Active standing tests in pediatric studies found symptom provocation rates ranging from more than one-third to nearly one-half, although time since injury varied.^9,11,15^ Importantly, the minimal overlap between AD and symptom provocation suggests distinct underlying mechanisms, further supported by distinct clinical profiles. This finding underscores the need to explore orthostatic intolerance within the broader spectrum of postconcussion symptoms. Contributing factors such as vestibular dysfunction, anxiety, and cervical spine injury likely play a role, emphasizing the importance of understanding the interplay to improve diagnosis, guide management, and optimize recovery.

The higher symptom burden observed in patients reporting symptom provocation, despite no association with objective AD, suggests that provocation may indicate broader concussion effects rather than underlying AD alone. This dissociation could reflect general physiological stress response postconcussion, heightened symptom sensitivity or altered interoception, or a disruption in other physiological systems that influence comfort. Psychological symptoms such as anxiety and depression may increase sensitivity to somatic sensations, as supported by evidence of elevated anxiety^29^ and catastrophizing in adolescents with POTS.^30^ Moreover, impaired vestibulo-ocular reflex, balance control issues, visual motion sensitivity, and cervicogenic symptoms are common postconcussion and may contribute to dizziness or nausea during unsupported postural changes. Altered systems may act independently or interactively, amplifying symptoms. Although not assessed herein, these potential mechanisms underscore the importance of comprehensive clinical assessment.

Interestingly, unlike the predominance of females in POTS,^17^ we observed no significant female predominance in AD after concussion. This finding is consistent with a study of patients aged 8 to 25 years presenting to a specialty clinic for concussion that found a similar prevalence of orthostatic tachycardia in the sexes.^14^ These findings suggest that the pathophysiology of postconcussion orthostatic tachycardia may differ from that of POTS.

### Clinical Implications

The distinction between physiological AD and symptom provocation enables targeted interventions. AD patients may benefit from autonomic support strategies, such as hydration, dietary modifications, and compression stockings,^31,32^ while those with symptom provocation may require multidisciplinary approaches addressing vestibular dysfunction, cervical spine involvement, visual disturbances, and mental health comorbidities (eg, vestibular rehabilitation, cervical physiotherapy, vision therapy, psychological counseling or cognitive-behavioral therapy, and psychosocial approaches^30^). Early physical activity and graded exercise programs can reduce concussion symptoms^33,34,35,36,37^ while addressing both AD and symptom provocation.^32,38^

The independence of symptom provocation from physiological AD questions current assessment approaches, especially in younger children whose self-reported symptom accuracy may be limited despite staff efforts to ensure comprehension (eg, blurry vision). This underscores the importance of a dual assessment approach combining objective measures with age-appropriate patient-reported outcomes for comprehensive and developmentally sensitive care.

The Concussion in Sport Group recently recommended assessing AD characterized by orthostatic intolerance,^39^ a known risk factor for prolonged symptoms.^40^ Understanding AD within the broader context of concussion symptoms is crucial to refining its role in protracted recovery and developing effective early interventions.

### Strengths and Limitations

Key strengths include the large sample size; the inclusion of both sport-related and non–sport-related concussions and younger children; the combination of objective measures with subjective symptom reports, offering a comprehensive assessment of AD; and the use of a simple, replicable clinical test to enhance applicability.

Limitations must also be acknowledged. First, the retrospective design used data from a specialty clinic serving a less acute and more refractory concussion population compared with emergency department samples, potentially limiting the generalizability of findings to broader populations. Although assessments were allowed up to 45 days postinjury, the actual time since injury was relatively narrow, reducing variability but introducing possible bias related to clinical referral patterns and patterns. Second, as a pragmatic (ie, observational) study relying on medical records, information on previous autonomic disorders (eg, POTS diagnosis), dietary habits, salt intake, and hydration was unavailable, and no previsit instructions were provided to patients. Third, 293 patients were excluded for not completing the orthostatic testing; while this was primarily due to clinical workflow constraints, reasons for noncompletion (eg, time limitations or inability to stand safely) were not systematically documented, limiting characterization of missingness. Fourth, reliance on a single standing time point without standardized timing or movement control introduces potential variability. Fifth, the absence of a control group precludes attributing AD prevalence or symptom provocation specifically to concussion-related factors. Sixth, given the limited number of patients meeting criteria for AD or reporting symptom provocation, findings from association analyses are best viewed as preliminary, hypothesis-generating, and intended to inform future research.

## Conclusions

This cohort study found that 10% of pediatric concussion patients showed objective AD, while 25% reported symptom provocation during postural change, with minimal overlap between the patients and distinct clinical profiles, challenging current clinical assumptions and suggesting that symptom provocation reflects general concussion symptom burden rather than underlying AD. Current findings emphasize the need for comprehensive evaluation beyond symptom reporting and indicate that reliance on a single physiological marker underestimates AD prevalence. Future studies should employ standardized protocols, diverse populations, and appropriate comparison groups to better understand associations of concussion with autonomic function.

## Notes

Supplement 1. **eFigure.** Flowchart of Patient Inclusion in the Study **eTable 1.** Incidence of Autonomic Dysregulation Characterized by Physical Signs (Tachycardia, Hypotension) or Symptom Provocation Upon Postural Change **eTable 2.** Orthostatic Vitals by Age Group **eTable 3.** Orthostatic Vitals By Autonomic Dysregulation (AD) Status

Supplement 2.
Nonauthor Collaborators

Supplement 3.
Data Sharing Statement
