# Error in Byline

**DOI:** 10.1001/jamanetworkopen.2025.34593

**Published:** 2025-08-29

In the Original Investigation titled “Prevalence of Orthostatic Autonomic Dysregulation in Pediatric Concussion,”^1^ published July 22, 2025, there was an error in the byline. Author Ewa Sucha’s credential was listed as PhD and should have been listed as MSc. This article has been corrected.^1^

## References

[1] Sicard V, Irani T, Ledoux A, . Prevalence of Orthostatic Autonomic Dysregulation in Pediatric Concussion. JAMA Netw Open. 2025;8(7):e2522309. doi:10.1001/jamanetworkopen.2025.22309 40694349 PMC12284742

