# Supplementary material for: Prevalence of Orthostatic Autonomic Dysregulation in Pediatric Concussion
Source: JAMA Netw Open. 2025 Jul 22;8(7):e2522309. doi: 10.1001/jamanetworkopen.2025.22309 (PMC12284742; doi:10.1001/jamanetworkopen.2025.22309)
Supplement: Supplement 1. — eFigure. Flowchart of Patient Inclusion in the Study eTable 1. Incidence of Autonomic Dysregulation Characterized by Physical Signs (Tachycardia, Hypotension) or Symptom Provocation Upon Postural Change eTable 2. Orthostatic Vitals by Age Group eTable 3. Orthostatic Vitals By Autonomic Dysregulation (AD) Status [file jamanetwopen-e2522309-s001.pdf]

Supplemental Online Content

Sicard V, Irani T, Ledoux AA, et al. Prevalence of orthostatic autonomic dysregulation in pediatric concucssion. *JAMA Netw. Open*. 2025;8(7):e2522309.
doi:10.1001/jamanetworkopen.2025.22309

eFigure. Flowchart of Patient Inclusion in the Study
eTable 1. Incidence of Autonomic Dysregulation Characterized by Physical Signs (Tachycardia, Hypotension) or Symptom Provocation Upon Postural Change
eTable 2. Orthostatic Vitals by Age Group
eTable 3. Orthostatic Vitals By Autonomic Dysregulation (AD) Status

This supplemental material has been provided by the authors to give readers additional information about their work.

© 2025 Sicard V et al. *JAMA Network Open*.

eFigure. Flowchart of Patient Inclusion in the Study

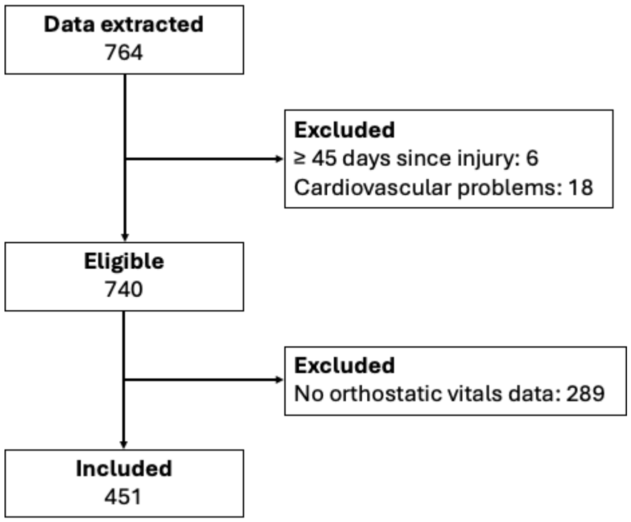

© 2025 Sicard V et al. *JAMA Network Open*.

eTable 1. Incidence of Autonomic Dysregulation Characterized by Physical Signs (Tachycardia, Hypotension) or Symptom Provocation Upon Postural Change

**Sample**	**n**	**Wilson score (%)**	**95% CI**	
			**Lower limit**	**Upper limit**
**Physical signs**				
Full sample	451	9.98	7.54	13.09
Boys aged 5-11	68	7.35	3.18	16.09
Boys aged 12-18	152	15.13	10.30	21.68
Girls aged 5-11	61	11.48	5.67	21.84
Girls 12-18	170	5.88	3.23	10.49
**Symptoms**				
Full sample	451	23.15	19.47	27.28
Boys aged 5-11	68	16.67	9.57	27.43
Boys aged 12-18	152	19.33	13.81	26.39
Girls aged 5-11	61	26.67	17.13	39.01
Girls 12-18	170	27.81	21.61	35.00

Notes. Determination autonomic dysregulation was based on the presence of at least one of these three symptoms upon standing: drop in systolic BP≥20 mmHg, drop in diastolic BP≥10 mmHg, or increase in HR≥40 bpm.x

© 2025 Sicard V et al. *JAMA Network Open*.

eTable 2. Orthostatic Vitals by Age Group

Characteristic, Median [IQR]	Full sample (N=451)	5-11 years old (n=129)	12-18 years old (n=322)
Supine SBP (mmHg)	114.00 (105.50, 123.00)	107.00 (100.00, 115.00)	117.00 (108.00, 125.00)
Standing SBP (mmHg)	113.00 (106.00, 122.00)	109.00 (103.00, 116.00)	115.00 (108.00, 124.00)
Orthostatic Change in SBP (mmHg)	0.00 (-6.00, 6.00)	1.00 (-4.00, 7.00)	-1.00 (-7.00, 6.00)
Supine DBP (mmHg)	65.00 (61.00, 70.00)	63.00 (60.00, 67.00)	67.00 (62.00, 70.00)
Standing DBP (mmHg)	72.00 (67.00, 77)	70.00 (66.00, 75.00)	73.00 (68.00, 78.00)
Orthostatic Change in DBP (mmHg)	7.00 (3.00, 11.00)	7.00 (3.00, 12.00)	7.00 (2.00, 11.00)
Supine HR (bpm)	69.00 (60.00, 77.00)	73.00 (66.00, 80.00)	67.00 (58.00, 75.00)
Standing HR (bpm)	88.00 (76.00, 98.00)	88.00 (77.00, 98.00)	86.5 (75.00, 97.75)
Orthostatic Change in HR (bpm)	18.00 (9.00, 27.00)	15.00 (7.00, 25.00)	18.00 (10.00, 28.00)

Notes. bpm=beats per minute; DBP=Diastolic Blood Pressure; HR=Heart Rate; mmHg=millimeters of mercury; SBP=Systolic Blood Pressure.

© 2025 Sicard V et al. *JAMA Network Open*.

eTable 3. Orthostatic Vitals by Autonomic Dysregulation (AD) Status

Characteristic, Median [IQR]	Full sample (N=451)	AD (n=45)	No AD (n=406)
Supine SBP (mmHg)	114.00 (105.50, 123.00)	65.00 (58.00, 71.00)	69.00 (60.00, 77.00)
Standing SBP (mmHg)	113.00 (106.00, 122.00)	99.00 (86.00, 110.00)	87.00 (75.00, 96.00)
Orthostatic Change in SBP (mmHg)	0.00 (-6.00, 6.00)	37.00 (20.00, 43.00)	17.00 (9.00, 25.00)
Supine DBP (mmHg)	65.00 (61.00, 70.00)	122.00 (110.00, 130.00)	113.00 (105.00, 122.00)
Standing DBP (mmHg)	72.00 (67.00, 77.00)	112.00 (103.00, 117.00)	114.00 (107.00, 122.00)
Orthostatic Change in DBP (mmHg)	7.00 (3.00, 11.00)	-14.00 (-23.00, -5.00)	1.00 (-5.00, 7.00)
Supine HR (bpm)	69.00 (60.00, 77.00)	66.00 (62.00, 72.00)	65.00 (61.00, 69.00)
Standing HR (bpm)	88.00 (76.00, 98.00)	70.00 (64.00, 78.00)	72.00 (68.00, 77.00)
Orthostatic Change in HR (bpm)	18.00 (9.00, 27.00)	2.00 (-7.00, 11.00)	7.00 (3.00, 11.00)

Notes. bpm=beats per minute; DBP=Diastolic Blood Pressure; HR=Heart Rate; mmHg=millimeters of mercury; SBP=Systolic Blood Pressure. The presence of orthostatic tachycardia or hypotension determined Autonomic Dysregulation (AD) status.

© 2025 Sicard V et al. *JAMA Network Open*.
