# Supplementary material for: Prevalence of Orthostatic Autonomic Dysregulation in Pediatric Concussion
Source: JAMA Netw Open. 2025 Jul 22;8(7):e2522309. doi: 10.1001/jamanetworkopen.2025.22309 (PMC12284742; doi:10.1001/jamanetworkopen.2025.22309)
Supplement: Supplement 2. — Nonauthor Collaborators [file jamanetwopen-e2522309-s002.pdf]

Supplemental Online Content: Nonauthor Collaborators
*First name, last name, and suffix (if applicable) are required and will appear in PubMed.

*Group Name(s): uOttawa Brain-Heart Interconnectome (BHI) and TRANSCENDENT Concussion Integrated Discovery Programs							
***First Name and Middle Initial(s)**	***Last Name**	***Suffix (eg, Jr, III)**	Academic Degrees	Institution	Location (city, state/province, country)	Role or Contribution, eg, chair, principal investigator	Group (if more than 1 Group listed in the byline) and/or Subgroup (eg, Steering Committee)
Noah	Silverberg		PhD	Faculty of Medicine, The University c	Vancouver, British Columb	Co-Principal Investigator	
Keith Owen	Yeates		PhD	Alberta Children's Hospital Research	Calgary, Alberta, Canada	Co-Principal Investigator	
Monica	Lamoureux		MSc	Children's Hospital of Eastern Ontari	Ottawa, Ontario, Canada	Director of Research Operations	
