# Supplementary material for: Prevalence of Orthostatic Autonomic Dysregulation in Pediatric Concussion
Source: JAMA Netw Open. 2025 Jul 22;8(7):e2522309. doi: 10.1001/jamanetworkopen.2025.22309 (PMC12284742; doi:10.1001/jamanetworkopen.2025.22309)
Supplement: Supplement 3. — Data Sharing Statement [file jamanetwopen-e2522309-s003.pdf]

Data Sharing Statement

Sicard. Prevalence of Orthostatic Autonomic Dysregulation in Pediatric Concussion. *JAMA Netw Open*. Published July 22, 2025. doi:10.1001/jamanetworkopen.2025.22309

Data
Data available: Yes
Data types: Deidentified participant data
How to access data: rzemek@cheo.on.ca
When available: With publication

Supporting Documents
Document types: None

Additional Information
Who can access the data: Data requests will go though Ontario Brain Institute Brain Code
Types of analyses: We will upload the deidentified data into OBI Brain-CODE as a controlled data release.
Mechanisms of data availability: https://www.braincode.ca/content/controlled-data-releases
